# Supplementary material for: Mind the gap: Data availability, accessibility, transparency, and credibility during the COVID-19 pandemic, an international comparative appraisal
Source: PLOS Glob Public Health. 2023 Apr 21;3(4):e0001148. doi: 10.1371/journal.pgph.0001148 (PMC10120928; doi:10.1371/journal.pgph.0001148)
Supplement: S1 Table — (DOCX) [file pgph.0001148.s001.docx]

S1 Table: List of all official and unofficial sources of information used, by country

| Bangladesh | - <http://dashboard.dghs.gov.bd/webportal/pages/covid19.php> - <http://103.247.238.92/webportal/pages/covid19-vaccination-update.php> - <http://103.247.238.92/webportal/pages/covid19-bedstatus.php> - <https://idare.io> - <https://archive.dhakatribune.com/bangladesh/2021/06/22/icddr-b-study-covid-19-infection-rate-higher-in-slum-areas> - <https://www.riiopenjournals.com/index.php/society_sustainability/article/view/283/105> - <https://www.banglanews24.com/national/news/bd/789134.details> - <https://www.genderandcovid-19.org/bangladesh/> - <https://cxb-epi.netlify.app/#dashboard> - <https://www.tbsnews.net/coronavirus-chronicle/covid-19-bangladesh/how-prisons-bangladesh-keep-covid-19-check-251437> - <https://www.kalerkantho.com/online/national/2021/05/23/1035643> |
| --- | --- |
| Indonesia | - <https://covid19.go.id/peta-sebaran> - <https://www.kemkes.go.id/downloads/resources/download/Ketersediaan-Tempat-Tidur-RS-Covid19/BOR-RS-7-JULI-2021.pdf> - <https://corona.jakarta.go.id/id> - <https://vaksin.kemkes.go.id/#/vaccines> - <https://kabar24.bisnis.com/read/20210108/15/1340235/kasus-covid-19-pada-anak-usia-sekolah-paling-banyak-dari-siswa-sd> - <https://www.suara.com/news/2021/02/19/181837/508-pelajar-meninggal-karena-covid-19-kemendikbud-masih-rendah> - <https://tirto.id/menghitung-jumlah-nakes-yang-positif-dan-meninggal-akibat-corona-f4Z4> - <https://nakes.laporcovid19.org> - <https://www.cnbcindonesia.com/news/20210626153835-4-256186/sedih-idi-sebut-401-dokter-meninggal-dunia-karena-covid-19> - <https://covid19.kemenkumham.go.id> - <https://sulsel.kemenkumham.go.id/berita-kanwil/berita-upt/6639-110-wbp-lapas-kelas-iia-watampone-jalani-vaksinasi-covid-19> - <https://megapolitan.okezone.com/read/2021/07/15/338/2441354/753-warga-binaan-di-lapas-paledang-bogor-jalani-vaksinasi-covid-19> - <https://megapolitan.kompas.com/read/2021/06/23/17392351/846-penghuni-lapas-dan-rutan-di-jakarta-jalani-vaksinasi-covid-19> - <http://dinkes.pamekasankab.go.id/detailpost/101-warga-binaan-pemasyarakatan-lapas-narkotika-iia-pamekasan-mendapat-suntikan-vaksin-covid-19> - <https://www.kompas.tv/article/184611/ratusan-warga-binaan-lapas-gorontalo-disuntik-vaksin-covid-19> - <https://www.kemkes.go.id/downloads/resources/download/Ketersediaan-Tempat-Tidur-RS-Covid19/BOR-RS-17-JULI-2021.pdf> - <https://metro.tempo.co/read/1414465/klaster-penjara-ratusan-petugas-dan-tahanan-rutan-jambe-positif-covid-19> - <https://nasional.kompas.com/read/2021/04/26/19591641/kpai-klaster-covid-19-sekolah-muncul-setelah-gelar-pembelajaran-tatap-muka?page=all> - <https://www.cnnindonesia.com/ekonomi/20201228123215-92-586955/waspada-klaster-pabrik-puluhan-ribu-buruh-positif-corona> |
| Nigeria | - <https://covid19.who.int/region/afro/country/ng> - <https://covid19.ncdc.gov.ng/> - <https://ourworldindata.org/covid-vaccinations?country=NGA> |
| Panama | - <https://rainforestfoundation.org/covid-panama-gis-map/> - <https://dtm.iom.int/reports/panama-seguimiento-la-emergencia-estaciones-de-recepci%C3%B3n-migratoria-pandemia-covid-19-5> - <https://www.eluniversal.com.mx/mundo/reportan-al-menos-390-contagios-tras-brote-de-covid-19-en-carcel-de-panama> - [Donations and important contributions: http://minsa.b-cdn.net/sites/default/files/publicacion-general/inventario_de_donacion_de_covid_06-2020.pdf](http://minsa.b-cdn.net/sites/default/files/publicacion-general/inventario_de_donacion_de_covid_06-2020.pdf) - <http://minsa.b-cdn.net/sites/default/files/publicacion-general/informe_covid19_4_de_julio_2021_presidencia.pdf> - <https://covid19.who.int/> |
| Netherlands | - [https://coronadashboard.rijksoverheid.nl](https://coronadashboard.rijksoverheid.nl/) - <https://ourworldindata.org/coronavirus#explore-the-global-situation> - <https://coronadashboard.government.nl/> - <https://coronadashboard.government.nl/landelijk/ziekenhuizen-en-zorg> - <https://data.humdata.org/dataset/covid-19-vaccinations> - <https://www.statista.com/statistics/1109459/coronavirus-death-casulaties-by-age-in-netherlands/> - [https://www.rivm.nl](https://www.rivm.nl/) - <https://www.statista.com/statistics/1109473/coronavirus-death-casulaties-by-gender-in-netherlands/> - <https://www.statista.com/statistics/1109473/coronavirus-death-casulaties-by-gender-in-netherlands/> - <https://www.statista.com/statistics/1101612/coronavirus-cases-by-province-in-the-netherlands/> - [https://www.government.nl](https://www.government.nl/) - <https://www.prisonstudies.org/sites/default/files/resources/downloads/keeping_covid_out_of_prisons.pdf> - <https://knoema.com/atlas/Netherlands/topics/Health/Health-Service-Coverage/Hospital-beds> - [https://covid19-country-overviews.ecdc.europa.eu/#27_Netherlands](https://covid19-country-overviews.ecdc.europa.eu/#27_Netherlands ) |
| Greece | - <https://eody.gov.gr/wp-content/uploads/2021/07/covid-gr-daily-report-20210705.pdf> - <https://covid19.gov.gr/covid19-live-analytics/> - <https://emvolio.gov.gr/vaccinationtracker> - <https://eody.gov.gr/wp-content/uploads/2021/06/covid-gr-daily-report-20210630.pdf> - <https://eody.gov.gr/wp-content/uploads/2021/02/Surveillance-refugees-weekly-GR-2021-02.pdf> - <https://www.kathimerini.gr/society/561166306/okto-klines-meth-covid-kenes-sti-thessaloniki-45-stin-attiki/> - <https://www.iatronet.gr/article/100282/molis-6-klines-meth-covid-diathesimes-sta-nosokomeia-ths-attikhs> - <https://www.protothema.gr/greece/article/1033490/halkidiki/> - <https://www.ethnos.gr/greece/article/146920/koronoiospanoapo30kroysmataseghrokomeiostoperisteri> - <https://www.protothema.gr/greece/article/1111581/koronoios-41-krousmata-sto-nosokomeio-gennimatas/> - <https://www.naftemporiki.gr/story/1737278/fulakes-patras-entopistikan-15-krousmata-koronoiou-thetikos-kai-o-n-palaiokostas> |
| Turkey | - <https://covid19.saglik.gov.tr/EN-69532/general-coronavirus-table.html> - <https://covid19.saglik.gov.tr/Eklenti/39230/0/covid-19-weekly-situation-report---43pdf.pdf?_tag1=D3D202441F1F5165A33D16981E6544EF7FC0A32F> - <https://covid19.saglik.gov.tr/Eklenti/39502/0/covid-19-daily-situation-report-23112020pdf.pdf?_tag1=06C8012A6112A45F521E94C75B8ECF25C89050BF> - <https://www.toraks.org.tr/site/news/10240> - <https://www.bto.org.tr/pandemi-esitsizlikleri-derinlestirdi/> |
| England (UK) | - <https://coronavirus.data.gov.uk/> - <https://coronavirus.data.gov.uk/details/vaccinations> - <https://coronavirus.data.gov.uk/details/deaths> - <https://coronavirus.data.gov.uk/details/cases> - <https://www.england.nhs.uk/statistics/statistical-work-areas/covid-19-hospital-activity/> - <https://www.england.nhs.uk/statistics/wp-content/uploads/sites/2/2021/07/COVID-19-weekly-announced-vaccinations-01-July-2021.pdf> - <https://www.england.nhs.uk/statistics/statistical-work-areas/covid-19-vaccinations/> - <https://coronavirus.data.gov.uk/details/deaths> - <https://www.ons.gov.uk/peoplepopulationandcommunity/healthandsocialcare/conditionsanddiseases/articles/coronaviruscovid19latestinsights/vaccines> - <https://www.ons.gov.uk/peoplepopulationandcommunity/birthsdeathsandmarriages/deaths/datasets/deathsinvolvingcovid19bylocalareaanddeprivation> - <https://www.theguardian.com/society/2021/jan/18/almost-30-of-covid-patients-in-england-re-admitted-to-hospital-after-discharge-study> <https://www.medrxiv.org/content/10.1101/2021.01.15.21249885v1.full.pdf> - <https://www.ons.gov.uk/peoplepopulationandcommunity/healthandsocialcare/conditionsanddiseases/datasets/coronaviruscovid19infectionsurveydata> - <https://www.ons.gov.uk/peoplepopulationandcommunity/birthsdeathsandmarriages/deaths/articles/coronaviruscovid19relateddeathsbyethnicgroupenglandandwales/2march2020to15may2020> - [https://www.icnarc.org/Our-Audit/Audits/Cmp/Reports Report 2021-07-05](https://www.icnarc.org/Our-Audit/Audits/Cmp/Reports%20Report%202021-07-05) - <https://www.gov.uk/government/publications/weekly-statistics-for-nhs-test-and-trace-england-8-july-to-14-july-2021> - <https://www.ons.gov.uk/peoplepopulationandcommunity/healthandsocialcare/healthinequalities/datasets/covid19vaccinationratesandoddsratiosbysociodemographicgroup> - <https://www.bmj.com/content/371/bmj.m4225https://> - <https://www.gov.uk/government/publications/covid-19-review-of-disparities-in-risks-and-outcomes> - <https://www.runnymedetrust.org/uploads/Runnymede%20Covid19%20Survey%20report%20v3.pdf> - <https://www.gov.uk/government/publications/demographic-data-for-coronavirus-testing-england-28-may-to-26-august/demographic-data-for-coronavirus-covid-19-testing-england-28-may-to-26-august> - <https://backup.ons.gov.uk/wp-content/uploads/sites/3/2020/09/Coronavirus-COVID-19-Infection-Survey-characteristics-of-people-testing-positive-for-COVID-19-in-England-Septe.pdf> - <https://www.ons.gov.uk/peoplepopulationandcommunity/healthandsocialcare/conditionsanddiseases/datasets/coronavirusandthesocialimpactsoflongcovidonpeopleslivesingreatbritain> - <https://www.ons.gov.uk/peoplepopulationandcommunity/birthsdeathsandmarriages/deaths/datasets/ratesofdeathsinvolvingcovid19bydisabilitystatusenglandandwales> - <https://www.tuc.org.uk/research-analysis/reports/riddor-covid-and-under-reporting> - <https://www.ons.gov.uk/peoplepopulationandcommunity/healthandsocialcare/conditionsanddiseases/datasets/coronaviruscovid19infectionsinthecommunityinengland> - <https://www.theguardian.com/uk-news/2021/jan/23/uk-asylum-seekers-told-claims-at-risk-if-they-misbehav> - <https://www.ons.gov.uk/peoplepopulationandcommunity/healthandsocialcare/healthandwellbeing/datasets/coronavirusandhighereducationstudents> - <https://www.ons.gov.uk/peoplepopulationandcommunity/healthandsocialcare/healthcaresystem/adhocs/12536coronavirusanduniversitystudents3to8november2020greatbritain> - <https://www.ons.gov.uk/peoplepopulationandcommunity/healthandsocialcare/conditionsanddiseases/bulletins/covid19schoolsinfectionsurveyround4england/march2021> - <https://www.gov.uk/government/publications/hmpps-weekly-covid-19-data-26-april-2021> - <https://www.sps.gov.uk/Corporate/Information/covid19/covid-19-information-hub.asp> - <https://assets.publishing.service.gov.uk/government/uploads/system/uploads/attachment_data/file/909207/HMPPS_COVID19_WE_07082020_Pub_Doc.pdf> - <https://www.gov.uk/government/statistics/hmpps-covid-19-statistics-june-2021> - <https://www.theguardian.com/uk-news/2021/jul/25/more-than-50-died-in-home-office-asylum-seeker-accommodation-in-last-five-years>? - <https://www.ons.gov.uk/peoplepopulationandcommunity/birthsdeathsandmarriages/deaths/datasets/preexistingconditionsofpeoplewhodiedduetocovid19englandandwales> - <https://www.ucl.ac.uk/news/2021/mar/covid-19-death-rate-among-people-prison-three-times-higher-public> - <https://www.ons.gov.uk/peoplepopulationandcommunity/healthandsocialcare/conditionsanddiseases/datasets/coronaviruscovid19infectionsinthecommunityinengland> - <https://coronavirus.data.gov.uk/details/testing> - <https://analytics.phe.gov.uk/apps/chime/> - <https://www.england.nhs.uk/statistics/statistical-work-areas/covid-19-hospital-activity/> |
| Northern Ireland (UK) | - <https://coronavirus.data.gov.uk/> - <https://coronavirus.data.gov.uk/details/vaccinations> - <https://coronavirus.data.gov.uk/details/deaths> - <https://coronavirus.data.gov.uk/details/cases> - <https://www.health-ni.gov.uk/sites/default/files/publications/health/doh-db-070721.pdf> - <https://app.powerbi.com/view?r=eyJrIjoiZGYxNjYzNmUtOTlmZS00ODAxLWE1YTEtMjA0NjZhMzlmN2JmIiwidCI6IjljOWEzMGRlLWQ4ZDctNGFhNC05NjAwLTRiZTc2MjVmZjZjNSIsImMiOjh9> - <https://www.health-ni.gov.uk/sites/default/files/publications/health/Coronavirus-related-health-inequalities-report.pdf> - <https://covid-19.hscni.net/ni-covid-19-vaccinations-dashboard/> - <https://www.ons.gov.uk/peoplepopulationandcommunity/healthandsocialcare/conditionsanddiseases/articles/coronaviruscovid19latestinsights/vaccines> - <https://www.nisra.gov.uk/publications/covid-19-related-deaths-northern-ireland> - <https://www.bmj.com/content/371/bmj.m4225https://> - <https://www.runnymedetrust.org/uploads/Runnymede%20Covid19%20Survey%20report%20v3.pdf> - <https://www.ons.gov.uk/peoplepopulationandcommunity/healthandsocialcare/conditionsanddiseases/datasets/coronavirusandthesocialimpactsoflongcovidonpeopleslivesingreatbritain> - <https://www.tuc.org.uk/research-analysis/reports/riddor-covid-and-under-reporting> - <https://www.ons.gov.uk/peoplepopulationandcommunity/healthandsocialcare/conditionsanddiseases/datasets/coronaviruscovid19infectionsinthecommunityinengland> - <https://www.theguardian.com/uk-news/2021/jan/23/uk-asylum-seekers-told-claims-at-risk-if-they-misbehav> - <https://www.ons.gov.uk/peoplepopulationandcommunity/healthandsocialcare/healthcaresystem/adhocs/12536coronavirusanduniversitystudents3to8november2020greatbritain> - <https://www.gov.uk/government/publications/hmpps-weekly-covid-19-data-26-april-2021> - <https://www.sps.gov.uk/Corporate/Information/covid19/covid-19-information-hub.asp> - <https://www.justice-ni.gov.uk/articles/weekly-situation-reports-october-2015> - <https://assets.publishing.service.gov.uk/government/uploads/system/uploads/attachment_data/file/909207/HMPPS_COVID19_WE_07082020_Pub_Doc.pdf> - <https://www.gov.uk/government/statistics/hmpps-covid-19-statistics-june-2021> - <https://www.theguardian.com/uk-news/2021/jul/25/more-than-50-died-in-home-office-asylum-seeker-accommodation-in-last-five-years>? - <https://www.nisra.gov.uk/publications/covid-19-related-deaths-and-pre-existing-conditions-march-2020-february-2021> - <https://www.health-ni.gov.uk/news/half-million-vaccines-administered-northern-ireland> - <https://coronavirus.data.gov.uk/details/testing> |
| Scotland (UK) | - <https://coronavirus.data.gov.uk/> - https://www.gov.scot/publications/coronavirus-covid-19-data-for-scotland/ - <https://coronavirus.data.gov.uk/details/vaccinations> - <https://coronavirus.data.gov.uk/details/deaths> - <https://coronavirus.data.gov.uk/details/cases> - <https://public.tableau.com/app/profile/phs.covid.19/viz/COVID-19DailyDashboard_15960160643010/Overview> - <https://www.gov.scot/publications/coronavirus-covid-19-daily-data-for-scotland/> - <https://www.opendata.nhs.scot/dataset/covid-19-in-scotland> - <https://www.opendata.nhs.scot/dataset/covid-19-vaccination-in-scotland> - <https://www.gov.scot/publications/coronavirus-covid-19-additional-data-about-adult-care-homes-in-scotland/> - <https://public.tableau.com/app/profile/sg.eas.learninganalysis/viz/COVID19-SchoolsandChildcareInformation/Introduction> - <https://www.ons.gov.uk/peoplepopulationandcommunity/healthandsocialcare/conditionsanddiseases/articles/coronaviruscovid19latestinsights/vaccines> - <https://www.bbc.com/news/uk-scotland-57344826> - <https://publichealthscotland.scot/publications/covid-19-statistical-report/covid-19-statistical-report-21-july-2021/> - <https://publichealthscotland.scot/publications/covid-19-statistical-report/covid-19-statistical-report-3-march-2021/> - <https://www.bmj.com/content/371/bmj.m4225https://> - <https://www.runnymedetrust.org/uploads/Runnymede%20Covid19%20Survey%20report%20v3.pdf> - <https://publichealthscotland.scot/publications/covid-19-statistical-report/covid-19-statistical-report-21-july-2021/dashboard/> - <https://www.nrscotland.gov.uk/statistics-and-data/statistics/statistics-by-theme/vital-events/deaths/deaths-background-information/ethnicity-of-the-deceased-person> - <https://www.ons.gov.uk/peoplepopulationandcommunity/healthandsocialcare/conditionsanddiseases/datasets/coronavirusandthesocialimpactsoflongcovidonpeopleslivesingreatbritain> - <https://www.tuc.org.uk/research-analysis/reports/riddor-covid-and-under-reporting> - <https://www.ons.gov.uk/peoplepopulationandcommunity/healthandsocialcare/conditionsanddiseases/datasets/coronaviruscovid19infectionsinthecommunityinengland> - <https://www.theguardian.com/uk-news/2021/jan/23/uk-asylum-seekers-told-claims-at-risk-if-they-misbehav> - <https://www.ons.gov.uk/peoplepopulationandcommunity/healthandsocialcare/healthcaresystem/adhocs/12536coronavirusanduniversitystudents3to8november2020greatbritain> - <https://www.gov.uk/government/publications/hmpps-weekly-covid-19-data-26-april-2021> - <https://www.sps.gov.uk/Corporate/Information/covid19/covid-19-information-hub.asp> - <https://assets.publishing.service.gov.uk/government/uploads/system/uploads/attachment_data/file/909207/HMPPS_COVID19_WE_07082020_Pub_Doc.pdf> - <https://www.edinburghnews.scotsman.com/news/crime/first-inmate-scottish-prison-dies-covid-19-2535551> - <https://www.gov.uk/government/statistics/hmpps-covid-19-statistics-june-2021> - <https://www.theguardian.com/uk-news/2021/jul/25/more-than-50-died-in-home-office-asylum-seeker-accommodation-in-last-five-years>? - <https://www.nrscotland.gov.uk/files//statistics/covid19/covid-deaths-report-week-19.pdf> - <https://beta.isdscotland.org/media/9117/2021-06-02_sicsag_report.pdf> - <https://coronavirus.data.gov.uk/details/testing> |
| Wales (UK) | - <https://coronavirus.data.gov.uk/> - <https://coronavirus.data.gov.uk/details/vaccinations> - <https://coronavirus.data.gov.uk/details/deaths> - <https://coronavirus.data.gov.uk/details/cases> - <https://public.tableau.com/app/profile/public.health.wales.health.protection/viz/RapidCOVID-19virology-Public/Headlinesummary> - <https://www.ons.gov.uk/peoplepopulationandcommunity/healthandsocialcare/conditionsanddiseases/articles/coronaviruscovid19latestinsights/vaccines> - <https://www.ons.gov.uk/peoplepopulationandcommunity/birthsdeathsandmarriages/deaths/datasets/deathsinvolvingcovid19bylocalareaanddeprivation> - <https://www.bbc.com/news/uk-wales-52380643> - <https://statswales.gov.wales/Catalogue/Health-and-Social-Care/coronavirus-covid-19/testing-data-for-coronavirus-covid-19/cumulativenumberofantibodytestsandresultsauthorisedforcriticalwork> - <https://www.ons.gov.uk/peoplepopulationandcommunity/birthsdeathsandmarriages/deaths/articles/coronaviruscovid19relateddeathsbyethnicgroupenglandandwales/2march2020to15may2020> - [https://www.icnarc.org/Our-Audit/Audits/Cmp/Reports Report 2021-07-05](https://www.icnarc.org/Our-Audit/Audits/Cmp/Reports%20Report%202021-07-05) - <https://www.bbc.com/news/uk-wales-52380643> - <https://dhcw.nhs.wales/information-services/welsh-data-hub/covid-19-data-hub/> - <http://www2.nphs.wales.nhs.uk:8080/CommunitySurveillanceDocs.nsf/3dc04669c9e1eaa880257062003b246b/e61c928e715ece3180258680003449c3/$FILE/Wales%20COVID-19%20vaccination%20enhanced%20surveillance%20-%20equality%20report.pdf> - <https://www.bmj.com/content/371/bmj.m4225https://> - <https://www.runnymedetrust.org/uploads/Runnymede%20Covid19%20Survey%20report%20v3.pdf> - <https://www.ons.gov.uk/peoplepopulationandcommunity/healthandsocialcare/conditionsanddiseases/datasets/coronavirusandthesocialimpactsoflongcovidonpeopleslivesingreatbritain> - <https://www.ons.gov.uk/peoplepopulationandcommunity/birthsdeathsandmarriages/deaths/datasets/ratesofdeathsinvolvingcovid19bydisabilitystatusenglandandwales> - <https://www.tuc.org.uk/research-analysis/reports/riddor-covid-and-under-reporting> - <https://www.ons.gov.uk/peoplepopulationandcommunity/healthandsocialcare/conditionsanddiseases/datasets/coronaviruscovid19infectionsinthecommunityinengland> - <https://www.theguardian.com/uk-news/2021/jan/23/uk-asylum-seekers-told-claims-at-risk-if-they-misbehav> - <https://www.ons.gov.uk/peoplepopulationandcommunity/healthandsocialcare/healthcaresystem/adhocs/12536coronavirusanduniversitystudents3to8november2020greatbritain> - <https://www.gov.uk/government/publications/hmpps-weekly-covid-19-data-26-april-2021> - <https://www.sps.gov.uk/Corporate/Information/covid19/covid-19-information-hub.asp> - <https://assets.publishing.service.gov.uk/government/uploads/system/uploads/attachment_data/file/909207/HMPPS_COVID19_WE_07082020_Pub_Doc.pdf> - <https://www.gov.uk/government/statistics/hmpps-covid-19-statistics-june-2021> - <https://phw.nhs.wales/publications/publications1/placing-health-equity-at-the-heart-of-the-covid-19-sustainable-response-and-recovery-building-prosperous-lives-for-all-in-wales/> - <https://www.theguardian.com/uk-news/2021/jul/25/more-than-50-died-in-home-office-asylum-seeker-accommodation-in-last-five-years> - <https://www.ons.gov.uk/peoplepopulationandcommunity/birthsdeathsandmarriages/deaths/datasets/preexistingconditionsofpeoplewhodiedduetocovid19englandandwales> - <https://www.ucl.ac.uk/news/2021/mar/covid-19-death-rate-among-people-prison-three-times-higher-public> - <https://coronavirus.data.gov.uk/details/testing> |
