# Supplementary material for: Mind the gap: Data availability, accessibility, transparency, and credibility during the COVID-19 pandemic, an international comparative appraisal
Source: PLOS Glob Public Health. 2023 Apr 21;3(4):e0001148. doi: 10.1371/journal.pgph.0001148 (PMC10120928; doi:10.1371/journal.pgph.0001148)
Supplement: Attachment — Submitted filename: Response To Reviewers_FINAL.docx [file pgph.0001148.s002.docx]

We thank the reviewers for their insightful comments and helpful suggestions. Together with a fully revised version of the manuscript we answer to the reviewers point by point below.

**Reviewer #1:**

1. L149/150: Reconfirm data availability for partially vaccinated for Nigeria.

**At the time of the search our students were able to access data for partially vaccinated in Nigeria only through unofficial sources. The text in the manuscript has been corrected to reflect this.**

2. L155/156: 'the British ones' - Consistency in reporting on the UK. E.g. UK, Britain or England, Scotland and Wales. Or clarify preference at start.

**The terms are now used consistently, and England, Scotland Wales and Northern Ireland are referred to as countries in the UK**

3. L158: Information not always from official sources - This is debatable, not reliable and difficult to compare across countries. What are these other sources? Social media? Where data is not available from official sources can this be regarded as data for the country in context of this paper? This should be addressed. Suggestion is to include only officially available data.

**We have now added a definition of official and unofficial sources. We believe it is important to map also the unofficial sources, because it shows their role in the total counts for countries in the Global South, but also the importance of mapping special interests group that would have been unnoticed (i.e. the health workers or inmates), mainly in the Global North. In a way, part of the scope of this paper is also to highlight the importance of collaboration with local stakeholders in such unprecedented circumstances. We have now extended this reflection also in the discussion.**

4. L158-160: Nigeria reported on region (states), Number confirmed positive, number admitted, number discharged, number of deaths.

**Data for positive cases, discharged patients, and deaths were accurately reported by students during the mapping exercise and transposed in our manuscript. What emerged from the search is that more stratified data were missing or not accessible at the time of the research. Ultimately, our students were not able to locate data on patients hospitalised due to covid-19, although data for discharged patients were accessible. Based on the assumption that the students who undertook this activity are credible knowers of their contexts (**[**https://doi.org/10.1016/S2214-109X(21)00301-6**](https://doi.org/10.1016/S2214-109X(21)00301-6) **) we relied on the data they brought upon. However, the experience of students may have posed a limitation. We acknowledge this factor in the discussion section of our manuscript.**

5. Consistency should be maintained in reporting on findings to make easy for reader to follow

**Consistency of the paper has increased throughout.**

6. L184/185: How was credibility of data of official and unofficial sources rated as high?? This is quite subjective thus should be reconsidered.

**We purposely designed the questions on accessibility and credibility as to assess a subjective opinion. These are aimed at having an assessment by the people who know and understand the context, and therefore can roughly judge the credibility of a certain organisation, for example, because of previous exposure, or because of constructive and positive interactions with the authorities during the pandemic. People who have been attentive to the management of the pandemic in their countries will have formed an idea of whom to believe and whom not to believe. We therefore value their contribution as the knowers of their contexts (**[**https://doi.org/10.1016/S2214-109X(21)00301-6**](https://doi.org/10.1016/S2214-109X(21)00301-6) **). We think that this is important to complete the picture of COVID data at country level, in particular in places where fake news have attempted infiltrating or even dominated the discourse. We made more explicit in the text that these were subjective assessments based on previous exposure to the national discourse around COVID-19 pandemic. It is important for this exercise because it values the informal knowledge of the context, which would be impossible to judge by only accessing the websites remotely.**

7. Improved clarity needed in the discussion section. E.g. It is not always clear what was a finding from this study vs reference to another study.

**We have increased clarity of the discussion by stressing which consideration was immediately coming from our own results, and which from the scientific literature**

**<><><><><><><><><><><><><><><><><><><><><><><><><><><><><><><><><><><><><>**

**Reviewer #2:**

Thank you for giving me the opportunity to read this paper which talks about a timely topic.

Though the paper is written in a clear and structured way, I have a couple of comments:

- Method
  - It would be helpful to clarify why the authors chose those nine countries. Was it just random because of who participated in the summer school? The authors should also be consistent in talking about 9 or 12 countries.

**The sampling was indeed opportunistic, but this choice was based on the fact that we relied on the informal knowledge of the context of local people, as integral part of the assessment. These were all professional living or knowing very well the local context and in an ideal position of assessing the subjective variables under study (credibility, accessibility) (**[**https://doi.org/10.1016/S2214-109X(21)00301-6**](https://doi.org/10.1016/S2214-109X(21)00301-6) **). We have added these considerations into the method section.**

- I also wonder whether someone checked the data each student brought to the table. I think the methodology would have been stronger if each country had been researched by one person and checked/reviewed by another person to ensure accuracy. It does not appear to me that a second person reviewed the data (dual control principle). For example, how did you ensure that each student found all relevant data available in the respective country?

**Our rational choice was to maximise diversity, thus allowing individual students to extract data from their own country even if it was not possible to have someone else to double check the outcome. However, when possible, data where checked by supervisors who, collectively covered four languages: Greek, Italian, English, and Spanish. *Ad hoc* sessions were also designed to guide the students in the process of exploring potential sources of data, interpreting them correctly, and filling in the spreadsheet adequately. This is now added to the method section.**

- Another big issue is that I haven’t seen a table that lists all links for each country where the authors have their data from. Maybe they submitted this, but I don’t see it in the submitted manuscript, and I cannot trust the figures without knowing the link/data sources from the different countries.

**We are returning the manuscript with a supplementary file containing all the sources used by students during the process.**

- Focus on disparities based on ethnicity /inequalities in COVID-19 morbidity
  - I think the strength of the paper lies in the focus on the inequalities which can be read from the data. I think the author should mention this earlier on (already in the introduction and abstract) and make this issue the main focus of the paper

**We have now made the focus on inequalities more explicit in the introduction, thanks for this comment.**

- Data sharing versus privacy protection / surveillance
  - The authors demand more data sharing, but I believe they should also (at least briefly) discuss what this means for privacy protection/surveillance in addition to the trust issue they mentioned.

**We have added this point to the discussion.**
